# Xanthine oxidoreductase is required for genotoxic stress-induced NKG2D ligand expression and gemcitabine-mediated antitumor activity

**DOI:** 10.18632/oncotarget.11042

**Published:** 2016-08-03

**Authors:** Xiulong Xu, Geetha Rao, Yi Li

**Affiliations:** ^1^ Institute of Comparative Medicine, Yangzhou University, Jiangsu Province, Yangzhou 225009, P.R. China; ^2^ College of Veterinary Medicine, Yangzhou University, Jiangsu Province, Yangzhou 225009, P.R. China; ^3^ Department of Anatomy and Cell Biology, Rush University Medical Center, Chicago, IL 60612, USA; ^4^ Lester and Sue Smith Breast Center, Baylor College of Medicine, Houston, TX 77030, USA

**Keywords:** xanthine oxidoreductase, breast cancer, NKG2D ligand, uric acid, MAP kinase

## Abstract

MICA/B (the major histocompatibility antigen-related chain A and B) and Rae I are stress-inducible ligands for the immune-receptor NKG2D. Mechanisms by which genotoxic stress and DNA damage induce the expression of NKG2D ligands remain incompletely understood. Here, we report that inhibition of xanthine oxidoreductase (XOR) activity by allopurinol or inhibition of XOR expression by gene knockdown abrogated genotoxic stress-induced expression of MICA/B and Rae I in three tumor cell lines. XOR knockdown also blocked gemcitabine-mediated antitumor activity in an orthotopic syngeneic mouse model of breast cancer. As a rate-limiting enzyme in the purine catabolic pathway, XOR generates two end-products, uric acid and reactive oxygen species (ROS). ROS scavenging had an insignificant effect on genotoxic drug-induced MICA/B expression but modestly inhibited radiation-induced MICA/B expression. Exogenous uric acid (in the form of monosodium urate) induced MICA/B expression by activating the MAP kinase pathway. Allopurinol blocked genotoxic stress-induced MAP kinase activation. Our study provides mechanistic insights into genotoxic stress-induced activation of the MAP kinase pathway and suggests that XOR is required for genotoxic stress-induced NKG2D ligand expression and gemcitabine-mediated antitumor activity.

## INTRODUCTION

The ligands for the NKG2D immunoreceptor include one group of retinoic acid early transcript-1 (Rae-1), H60, and Mult1 in rodents, four UL-16 binding proteins and the MHC class I-related chain A and B (MICA/B) in humans. These ligands bind the NKG2D receptor expressed on natural killer cells, γδ T cells, and CD8^+^ T cells, and play an important role in activating innate immunity [1, 2]. MICA/B proteins are not expressed in normal epithelial cells except at low levels in variable areas of the intestinal epithelium of healthy individuals [3–5]. MICA/B expression is increased in melanoma, breast, colon, hepatocellular, and prostate cancers and some leukemias [4, 6–8], due in part to oncogene mutations [9–13]. For example, activation of the MAP kinase pathway by oncogene mutations such as RAS or BRAF induces MICA/B expression in thyroid tumor cell lines [12]. Ras activation induces the expression of murine NKG2D ligand expression [14]. DNA damage by exposure to radiation and genotoxic drugs induces NKG2D ligand expression in tumor and fibroblast cells [13, 15, 16]. Mechanistic studies suggest that ROS plays a critical role in DNA damage-induced NKG2D ligand expression in part through E2F transcriptionl regulation [17–20]. Alternatively, presence of DNA in the cytoplasm and activation of STING in DNA-damaged cells leads to the activation of IRF-3 transcription factor and induction of NKG2D ligand expression [21, 22].

Xanthine oxidoreductase (XOR) is a rate-limiting enzyme in the purine catabolic pathway [23]. Uric acid, one of its end products, has been identified as a novel “danger signal” that activates innate immunity [24–26]. Uric acid produced in apoptotic or necrotic cells induces the expression of the co-stimulatory molecules CD80 and CD86 in dendritic cells and promote dendritic cell maturation [24–26]. Recent studies demonstrated that XOR expression is decreased in several types of malignancy, including breast, colon, gastric, lung cancers [27–31]. How XOR expression contributes to an aggressive tumor behavior and a shorter survival remains unclear [28]. XOR is not only a differentiation marker but also plays an important role in promoting the inflammatory state [32]. Gibbings et al. [33] reported that XOR expression is increased in the inflammatory mononuclear phagocytes and plays an important role in CINC-1 (Cytokine-Induced Neutrophil Chemoattractant-1) secretion and differentiation of mononuclear phagocytes into the M2 phenotype of macrophages. Here we report that XOR plays a crucial role in activating the MAP kinase pathway and inducing NKG2D ligand expression in tumor cells undergoing genotoxic stress, and that XOR is required for gemcitabine-mediated antitumor activity in a syngeneic mouse breast cancer model.

## RESULTS

### Genotoxic stress leads to increased uric acid and ROS production

**Figure 1:**
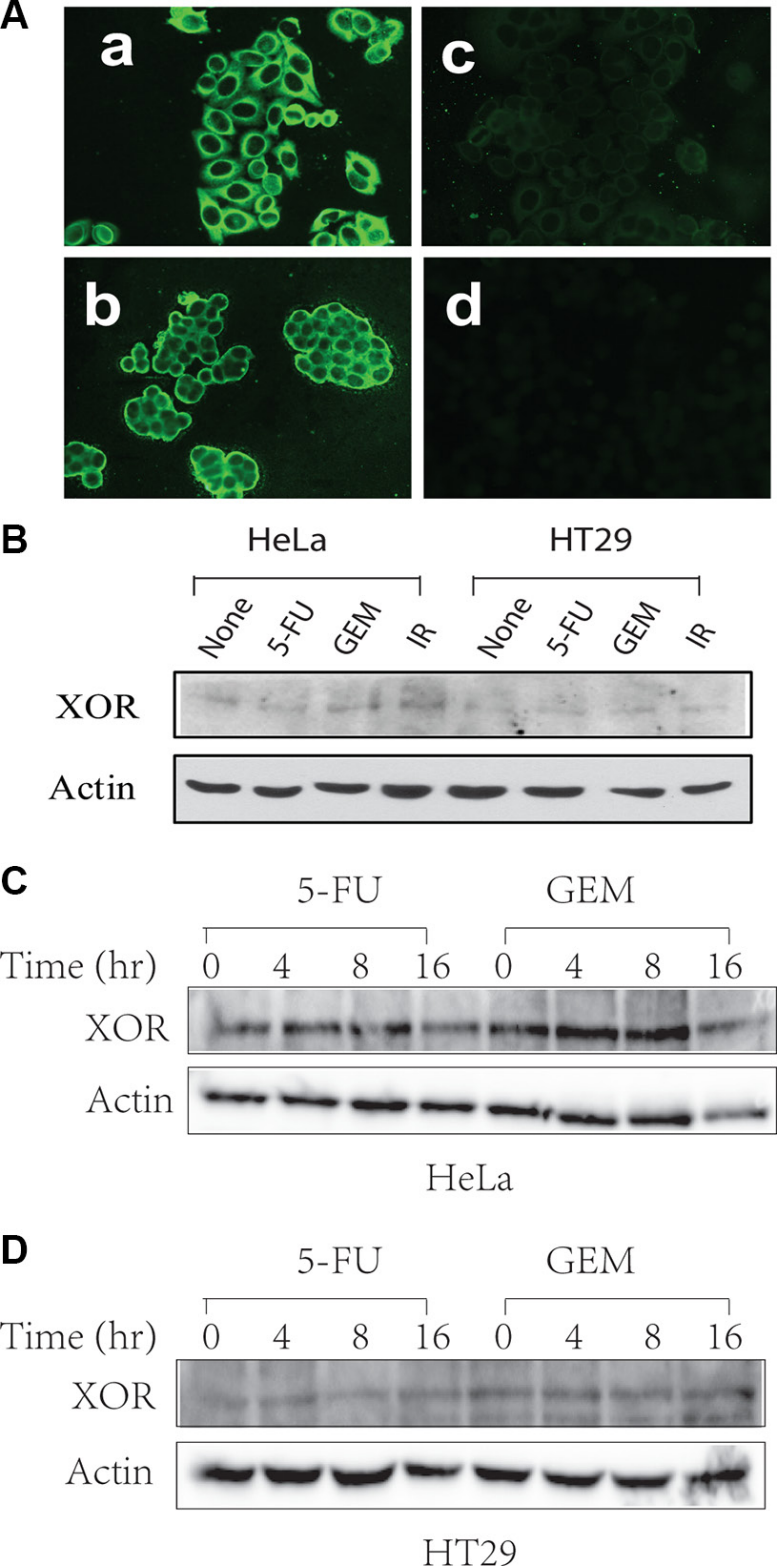
XOR expression in HeLa and HT29 cells (**A**) IF staining of XOR. HeLa (a) and HT29 (b) cells were analyzed for XOR expression by IF staining with an anti-XOR antibody. (c) The specificity of the antibody on HeLa cells was confirmed by pre-mixing anti-XOR antibody (1:50 in 200 μl) with purified XOR (2 μg/200 μl). Normal rabbit IgG was included as a negative control (d). (**B**–**D**) Genotoxic stress does not alter XOR expression. HeLa and HT29 cells were left untreated or treated with 5-FU (10 μM), gemcitabine (2 μM) or radiation (40 Gy) for 24 hr (B) or treated with 5-FU (10 μM) or gemcitabine (2 μM) in HeLa (C) and HT29 cells (D) for the indicated time. Cell lysates were prepared in NP-40 lysis buffer and analyzed for XOR expression by Western blot with a rabbit anti-XOR antiserum.

Uric acid is a novel “danger” signal that is capable of alerting the immune system [34]. XOR, a rate-limiting enzyme that catalyzes xanthine to produce uric acid and ROS, is differentially expressed in a variety of malignancies [27–30]. We first confirmed XOR expression in the cytoplasm of HeLa and HT29 cells by immunofluorescence staining (Figure 1A). Western blot analysis revealed the expression of XOR as an approximately 150-kDa protein. Genotoxic stress did not significantly alter the levels of XOR expression in HT29 and HeLa cells (Figure 1B). Furthermore, XOR levels did not change over different times in genotoxic drug-treated HT29 (Figure 1D) and HeLa cells (Figure 1C).

**Figure 2:**
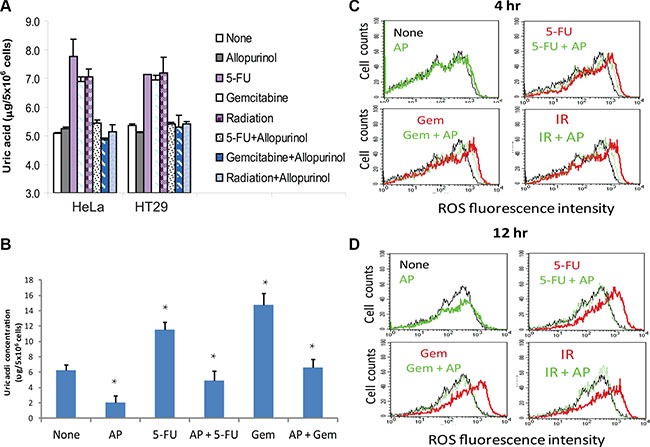
Allopurinol inhibits uric acid and ROS production (**A**) Uric acid production in HeLa and HT29 cells. Cells were left untreated or treated with 5-FU (10 μM), gemcitabine (2 μM) or radiation (40 Gy) in the absence or presence of allopurinol for 24 hr. Intracellular uric acid concentrations were determined by analyzing cell extracts with a uric acid kit. (**B**) Uric acid production in RCAS-Neu cells. Cells were left untreated or treated with 5-FU (10 μM) or gemcitabine (2 μM) in the absence or presence of allopurinol for 24 hr. Uric acid was analyzed as described in Materials and Methods. The data in (A) and (B) represent the mean + standard deviation in triplicate from one of three experiments with similar results. (**C** and **D**) ROS production. HT29 cells were similarly treated and analyzed for ROS production by FACS analysis of DCF-DA-loaded cells at 4 (C) or 12 (D) hr. Black line, no treatment; Red line, genotoxic stress treatment; Green line, allopuinol alone or plus genotoxic stress treatment.

Uric acid accumulates in tumor tissue following genotoxic drug treatment [35]. Here we tested if uric acid accumulated in HeLa and HT29 cells after genotoxic stress, including 5-FU, gemcitabine or radiation. As shown in Figure 2A, intracellular uric acid levels were elevated at least by 50% in two tumor cell lines after exposure. Allopurinol, an analog of hypoxanthine and a specific XOR inhibitor, completely blocked the accumulation of uric acid in the cells treated with 5-FU, gemcitabine, or radiation. Similar observation was made with RCAS-Neu, a murine breast cancer cell line (Figure 2B). Intracellular ROS levels were slightly elevated in HT29 cells when analyzed at 4 hr (Figure 2C) and significantly elevated 12 hr (Figure 2D) after exposure to 5-FU, gemcitabine, or radiation (red line) (Figure 2D), compared to the fluorescence level in untreated cells (black line) as a control. The shift of the histogram curve indicates the increase of ROS levels in cells treated with genotoxic drugs and radiation. Allopurinol had litter effect on inhibiting ROS production at 4 hr but completely inhibited ROS production (green line) at 12 hr after HT29 cells were exposed to 5-FU, gemcitabine, or radiation. Similar observations were made with HeLa cells (data not shown).

### XOR is required for genotoxic stress-induced MICA/B expression

**Figure 3:**
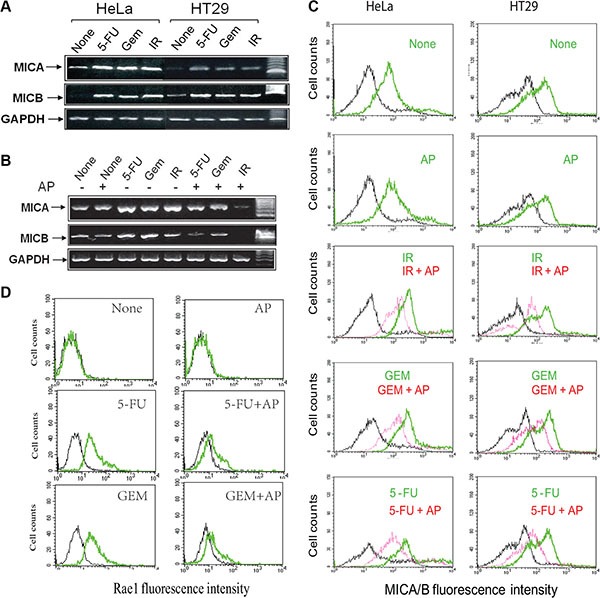
Allopurinol inhibits genotoxic stress-induced NKG2D ligand expression (**A**) HeLa and HT29 cells were left untreated, or treated with 5-FU (10 μM), gemcitabine (2 μM) or radiation (40 Gy) for 16 hr and analyzed for mRNA levels of MICA, MICB, and GAPDH by RT-PCT. (**B**) Allopurinol blocks genotoxic stress-induced MICA/B gene expression. HT29 cells were left untreated or treated with allopurinol (250 μg/ml) for 1 hr, followed by treatment with 5-FU (10 μM) and gemcitabine (2 μM) for 24 hr or radiation (40 Gy). Cells were harvested and analyzed for MICA/B and GAPDH mRNA by RT-PCT. (**C**) Allopurinol blocks genotoxic stress-induced MICA/B expression. HeLa and HT29 cells were treated as in Figure 3B and analyzed for cell surface MICA/B levels by FACS. Black line, isotype control; Green line, MICA/B in the cells treated with gemcitabine, 5-FU, or radiation alone; Red line, MICA/B in the cells treated with gemcitabine, 5-FU, or radiation in the presence of allopurinol. (**D**) Allopurinol blocks genotoxic stress-induced Rae I expression. RCAS-Neu cells were treated as in Figure 3B and analyzed for Rae I levels by FACS.

Genotoxic stress or DNA damage leads to increased NKG2D ligand expression [13, 36–39]. Consistent with these observations, we found that the levels of MICA and MICB transcripts were increased in HeLa and HT29 cells exposed to 5-FU (10 μM), gemcitabine (2 μM), and radiation (40 Gy) (Figure 3A). Allopuinol alone had no effect on MICA and MICB mRNA expression but blocked genotoxic stress-induced MICA and MICB mRNA expression (Figure 3B).

We next conducted FACS analysis to examine MICA/B expression. As shown in Figure 3C, untreated control HeLa and HT29 cells (marked as None) expressed modest levels of MICA/B (green line). Antibody isotype control was shown in a black line in all histograms. 5-FU, gemcitabine (GEM), or radiation (IR) significantly increased MICA/B expression, as shown by the green line representing MICA/B-positive cells that significantly shifted to right side, compared to untreated control. Allopurinol had no effect on basal level MICA/B expression but blocked MICA/B expression in the cells exposed to 5-FU, gemcitabine, or radiation (red line) (Figure 3C).

RCAS-Neu cells, a murine breast cancer cell line, did not express Rae I (labeled as a green line) (comparing with isotype control, labeled as a black line) (Figure 3D) (left upper histogram). Consistently, 5-FU (10 μM) and gemcitabine (2 μM) increased Rae I expression (Figure 3D, left middle and bottom histograms respectively). AP blocked 5-FU- and gemcitabine-induced Rae I expression in RCAS-Neu cells (Figure 3D).

**Figure 4:**
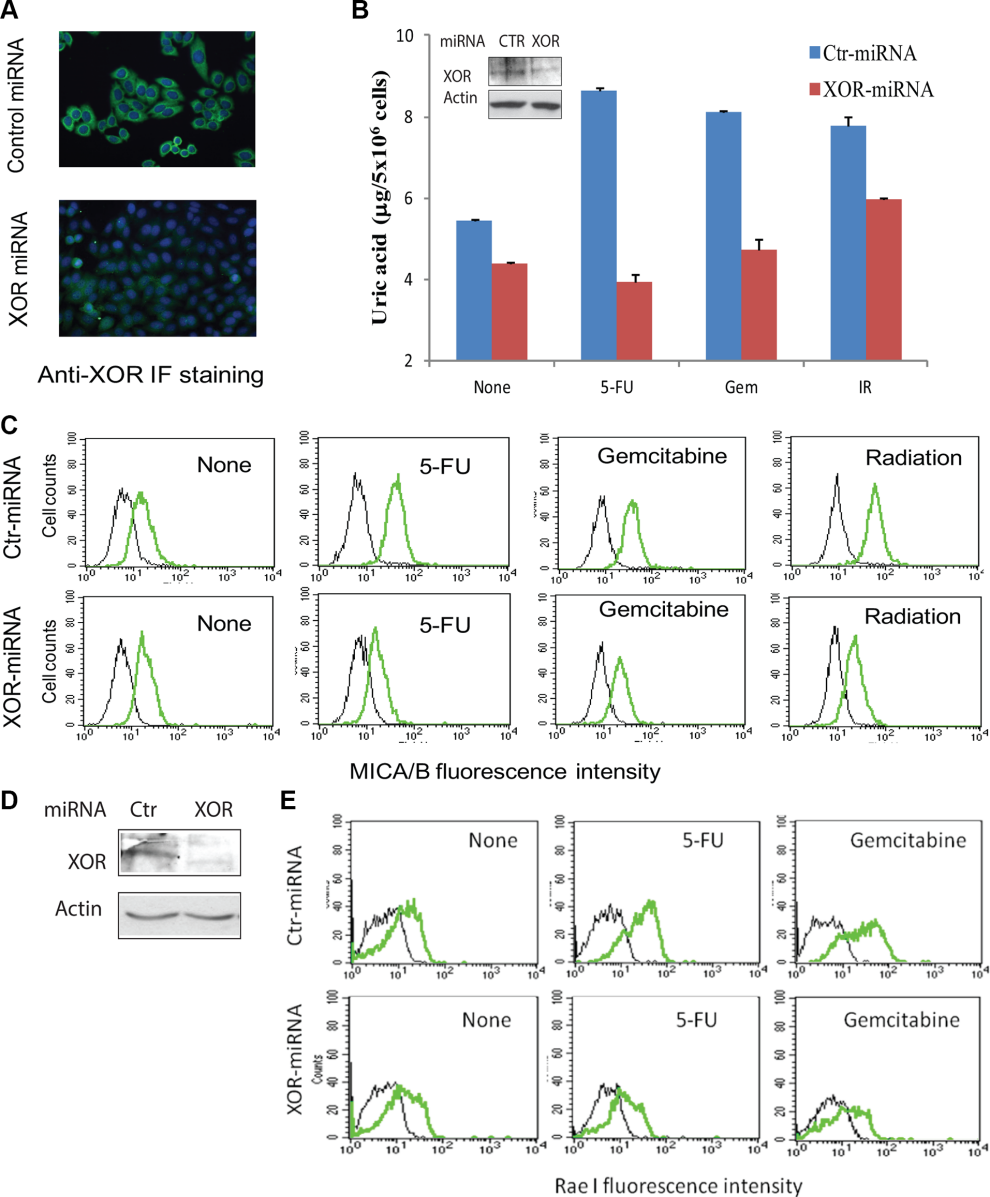
XOR suppression inhibits genotoxic stress-induced uric acid production and NKG2D ligand expression (**A**) Immunofluorescence (IF) staining of XOR. HeLa cells stably transfected with control miRNA (Ctr-miRNA) or XOR-miRNA were analyzed for XOR expression by IF staining with an anti-XOR antibody as described in Figure 1A. Nuclei was stained with DAPI. (**B** and **C**) XOR knockdown blocks uric acid production and MICA/B expression. Ctr-miRNA and XOR-miRNA-transfected cells were exposed to 5-FU (10 μM), gemcitabine (2 μM), or radiation (40 Gy). Cells were harvested at 24 hr and analyzed for intracellular uric acid levels using a uric acid assay kit (B) or for MICA/B expression by FACS (C). Black line, isotype control; Green line, MICA/B. Inset in (B): XOR expression analyzed by Western blot. Actin was included as a loading control. (**D**) Western blot analysis of XOR expression in Ctr-miRNA- and XOR-miRNA-transfected RCAS-Neu cells. (**E**) XOR knockdown blocks genotoxic stress-induced Rae I expression. Ctr-miRNA- and XOR-miRNA-transfected RCAS-Neucells were exposed to 5-FU (10 μM) or gemcitabine (2 μM) and analyzed for Rae I expression 16 hr later by FACS analysis.

To verify the role of XOR in mediating genotoxic stress-induced MICA/B expression in tumor cells, we tested if XOR suppression also blocked genotoxic stress-induced MICA/B expression. XOR expression was readily detectable by immunofluorescene staining in control-miRNA- but not in XOR- miRNA-transfected HeLa cells (Figure 4A). Suppression of XOR expression was confirmed by Western blot (Figure 4B, inset). Uric acid production (Figure 4B) and MICA/B expression (Figure 4C) were increased in control-miRNA- but not XOR-miRNA-transfected HeLa cells treated with 5- FU, gemcitabine, and radiation (Figure 4B). Similar results were observed with HT29 cells transfected with control-miRNA and XOR-miRNA (data not shown). Knockdown of XOR in RCAS-Neu cells, as revealed by Western blot (Figure 4D), also led to the suppression of genotoxic stress-induced Rae I expression (Figure 4E).

### Role of ROS in regulating MICA/B expression

**Figure 5:**
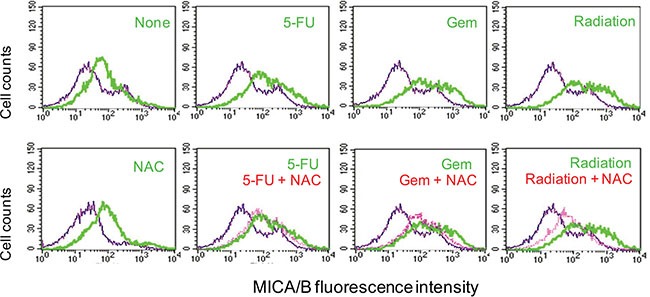
Effect of NAC on MICA/B expression HT29 cells were preincubated with NAC (10 mM) for 1 hr and then left unstimulated or stimulated with 5-FU (10 μM), gemcitabine (2 μM), or radiation (40 Gy). Cells were harvested 16 hr later and then analyzed by FACS for MICA/B expression. Blue line, isotype control; Green line, MICA/B; Red line, MICA/B in the cells treated with NAC plus 5-FU, gemcitabine or radiation.

We next tested whether induction of MICA/B expression in tumor cells under genotoxic stress was due to ROS production. N-acytelcysteine (NAC), an ROS scavenger, did not inhibit the induction of MICA/B expression in HT29 cells exposed to 5-FU or gemcitabine, but partially inhibited radiation-induced MICA/B expression in HT29 cells (Figure 5). NAC was able to completely scavenge ROS in HT29 cells exposed to 5-FU, gemcitabine or radiation (data not shown).

### Uric acid induces NKG2D ligand expression

**Figure 6:**
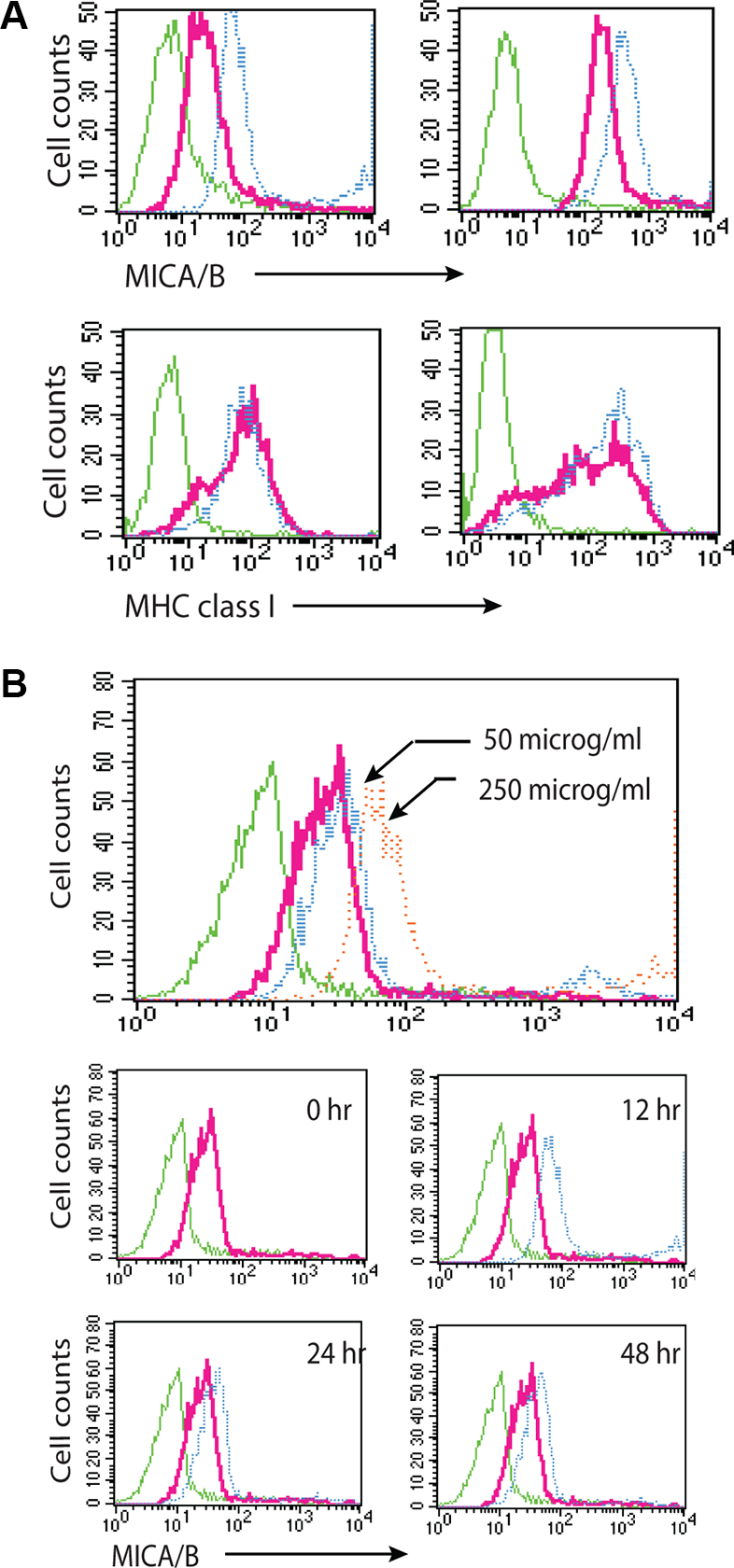
Uric acid induces MICA/B expression (**A**) Induction of MICA/B expression by MSU in HT29 and HeLa cells. Cells grown in 6-well plates were stimulated with MSU or allopurinol and stained for MICA/B and MHC class I expression. (**B**) Time course and dose response of induction of MIC/B expression by MSU. Induction of MICA/B expression by MSU in HT29 cells incubated for 16 hr with 50 or 250 μg/ml MSU (upper histogram) or with 250 μg/ml MSU for the indicated time (middle and bottom histograms). Green line, isotype control for untreated cells; Red line, MICA/B in unstimulated cells; Blue line, MICA/B in MSU-treated cells. There was no difference in the isotype fluorescence between untreated and MSU-treated cells. MICA/B fluorescence in MSU-treated cells was overlayed on that of untreated control for comparison.

We next tested whether exogenous uric acid was able to induce MICA/B expression. Due to its acidity and poor solubility [40], uric acid was used in its sodium form, monosodium urate (MSU). MICA/B expression was induced in HeLa and HT29 cells (Figure 6A, upper panel) exposed to MSU (blue line) for 16 hr, compared to untreated control (red line). Isotype control is shown in a green line). MSU did not induce MHC class I expression in either cell line (Figure 6A, bottom panel). HT29 cells treated with MSU (250 μg/ml) expressed more MICA/B at 12 hr than at 24 and 48 hr (Figure 6B, bottom panels).

### Uric acid-induced MICA/B expression is mediated by MAP kinase activation

**Figure 7:**
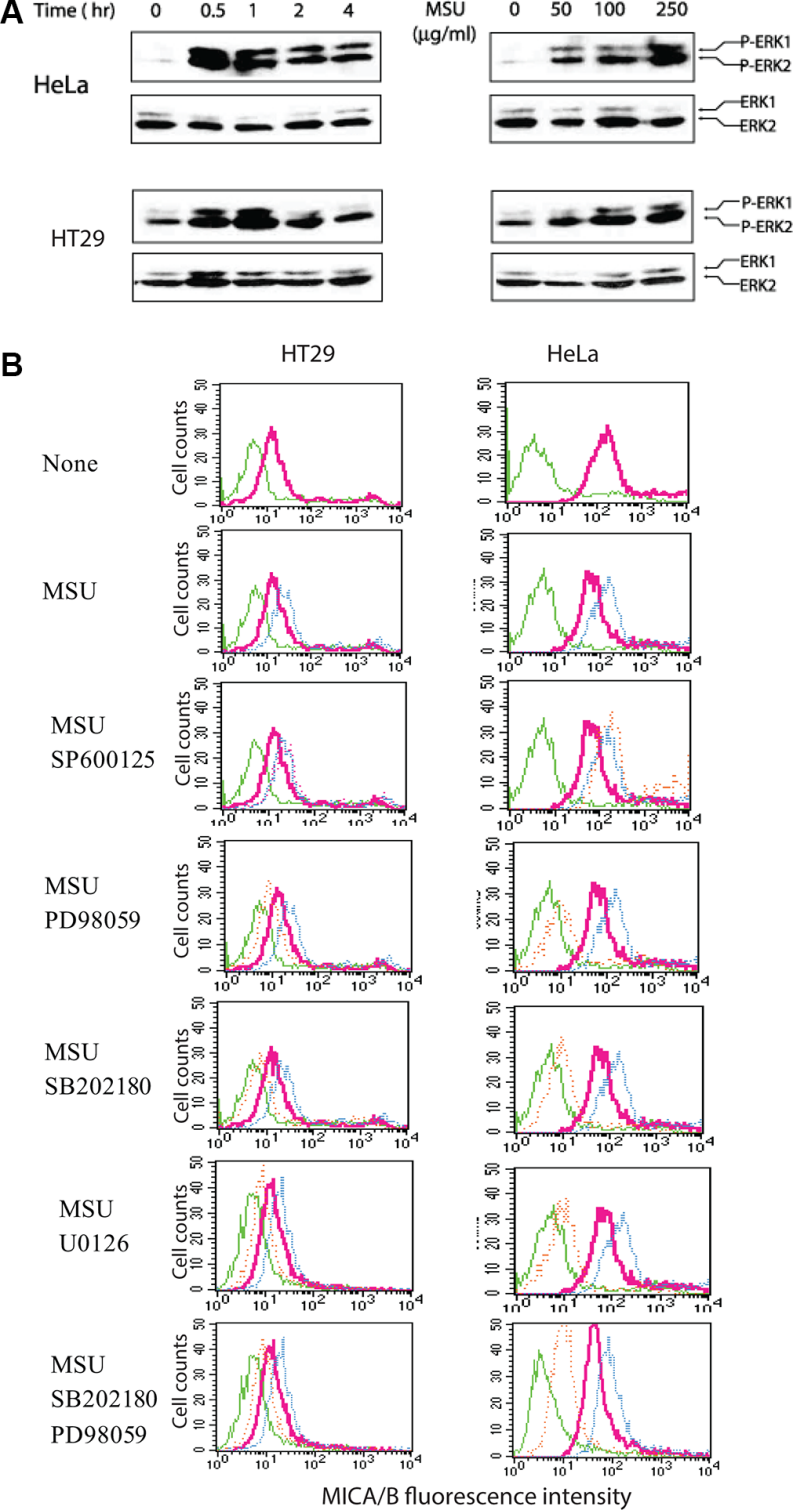
Activation of the MAP kinase pathway is required for uric acid-induced MICA/B expression (**A**) MSU induces ERK1/2 phosphorylation. HeLa and HT29 cells starved overnight in the serum-free medium were left unstimulated or stimulated with MSU (250 μg/ml) for the indicated time (left panel) or for 30 min with the indicated concentrations (right panel). Cells were harvested and analyzed for ERK1/2 phosphorylation by Western blot with an antibody against phosphorylated and total ERK1/2 (A). (**B**) Inhibition of MSU-induced MICA/B expression by MAP kinase inhibitors. HeLa and HT29 cells were left unstimulated or stimulated with MSU (250 μg/ml) in the absence or presence of the indicated inhibitors for 16 hr. Cells were harvested and analyzed for MICA/B expression by FACS. Green line, isotype control; Red line, MICA/B in the cells with no inhibitors; Blue line, MICA/B in the cells treated with MSU; Orange line, MICA/B in the cells treated with MSU plus the indicated inhibitors.

Constitutive MAPK activation due to oncogene mutations such as RAS and BRAF plays an important role in inducing MICA/B expression [12, 14, 41]. MSU dramatically induced ERK1/2 phosphorylation in HeLa and HT29 cells peaked at 30–60 min after MSU addition (Figure 7A, left panel) and in a dose-dependent manner (Figure 7A, right panel). PD98059 (50 μM) and U0126 (2 μM) inhibited the expression of MSU-induced MICA/B expression in HT29 and HeLa cells (Figure 7B). The inhibitor of p38 MAP kinase, SB202180 (2 μM), also inhibited MSU-induced MICA/B expression, though p38 activation was not detected in these two cell lines (data not shown), probably due to the low sensitivity of the antibody against p38. PD98059 plus SB202180 did not block MICA/B expression more than either inhibitor alone. SP600125 (20 μM), an inhibitor of JNK, did not inhibit MICA/B expression in MSU- stimulated HT29 and HeLa cells.

### Intracellular uric acid concentrations in MSU-treated cells

**Figure 8:**
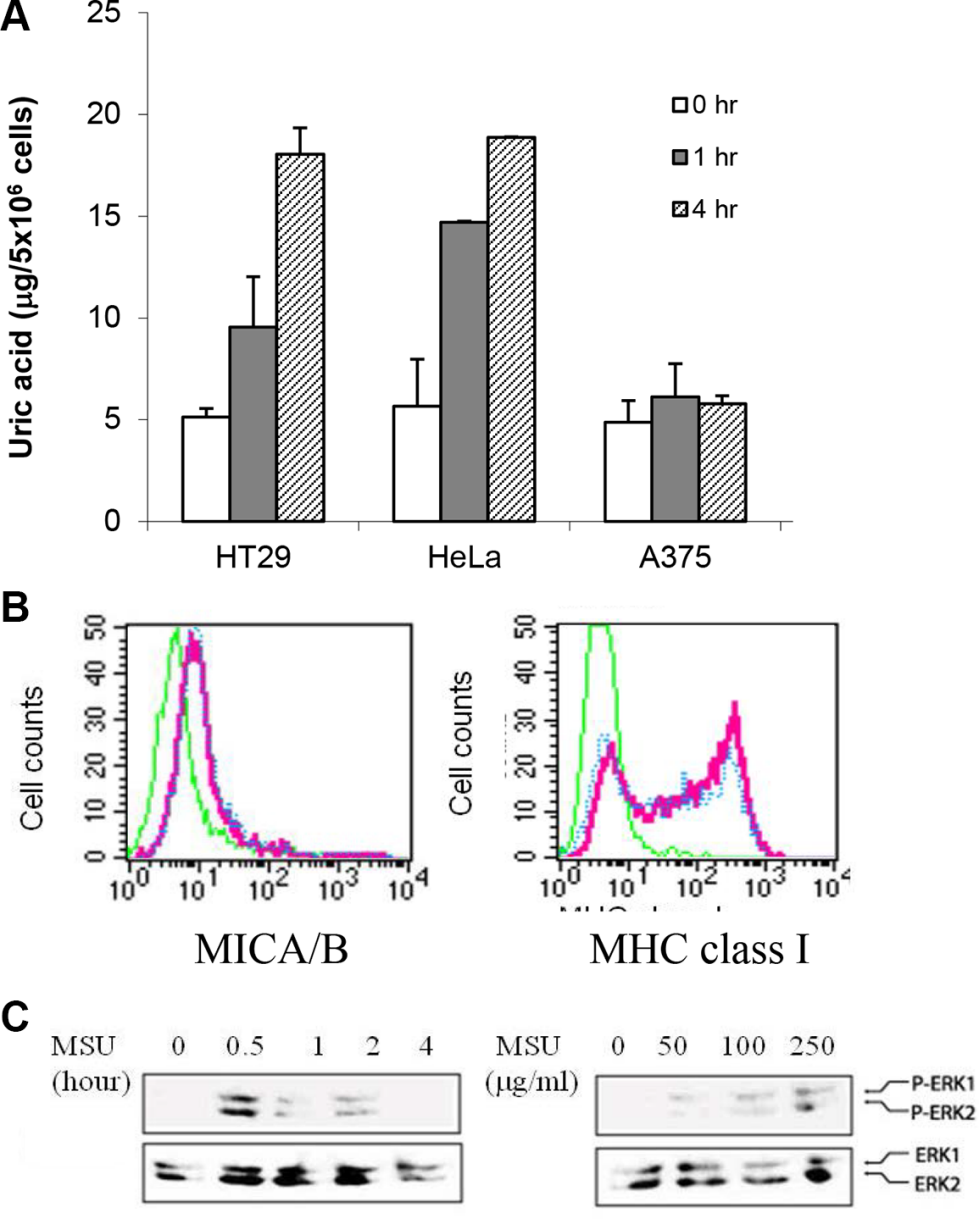
Increased intracellular uric acid levels in MSU-treated cells (**A**) Uric acid uptake. HeLa, HT29, and A375 cells were treated with MSU (250 mg/ml) for 1 or 4 hr. Cell extracts were analyzed for intracellular uric acid concentrations. (**B**) Inability of MSU to induce MICA/B expression in A375 cells. A375 cells were treated with MSU (250 mg/ml) for 24 hr and analyzed for MICA/B or MHC class I expression by FACS. (**C**) Inability of MSU to induce MAP kinase activation in A375 cells. A375 cells were treated with MSU (250 mg/ml) for 30 min and analyzed for MAP kinase activation by Western blot with anti-phospho and total ERK antibodies.

To determine if exogenous MSU used in our study was physiologically relevant, we tested if MSU was taken into the cytoplasm and reached the concentrations equivalent to that in the cells exposed to genotoxic stress. As shown in Figure 8A, intracellular uric acid concentrations reached up to 20 μg/5 × 10^6^ cells at 4 hr after MSU addition (250 μg/ml). Intracellular uric acid levels were increased in HT29 and HeLa cells but only slightly increased in A375 cells. Interestingly, MSU only slightly induced MICA/B expression (Figure 8B) and ERK1/2 phosphorylation (Figure 8C) in A375 cells.

### Uric acid accumulation precedes genotoxic stress-induced MICA/B expression and MAP kinase activation

**Figure 9:**
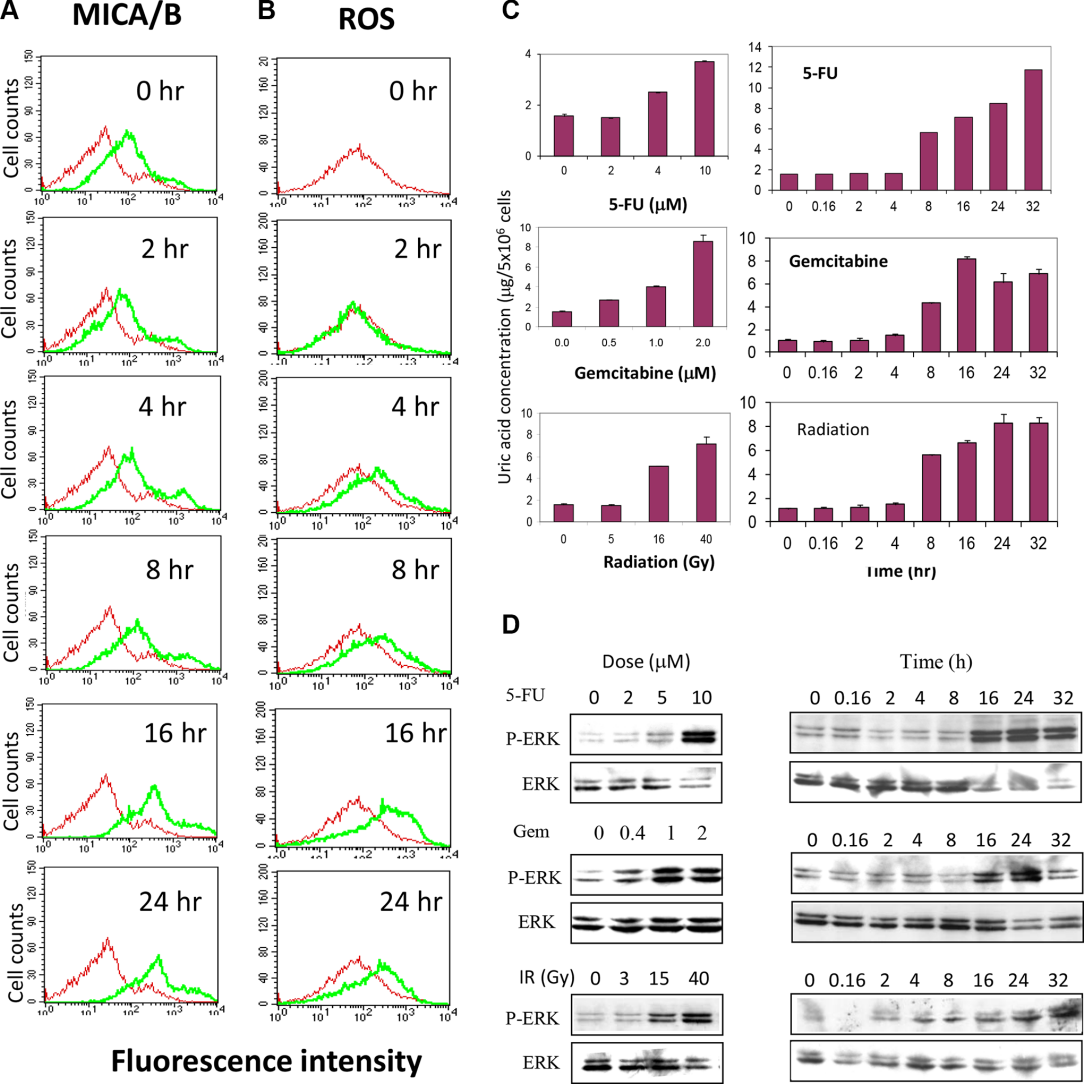
Temporal induction of MICA/B expression, MAP kinase activation, ROS and uric acid production HT29 cells seeded in 6-well plates were treated with gemcitabine (2 μM) for the indicated time. Cells were harvested and analyzed for MICA/B expression (**A**) or ROS production (**B**). (**C** and **D**) Dose- and time-dependent uric acid production and ERK phosphorylation. HT29 cells seeded in 6-well plates were left unstimulated or stimulated with the indicated concentrations of 5-FU, gemcitabine, or radiation for 20 hr or treated with 5-FU (10 μM) and gemcibatine (2 μM) or after irradiation (40 Gy) for the indicated time. Intracellular uric acid concentrations were determined by analyzing the cell extracts with a uric acid kit (C). MAP kinase activation was analyzed by Western blot with anti-phospho and total ERK1/2 antibodies (D).

To further investigate the role of uric acid production in mediating genotoxic stress-induced MICA/B expression, we conducted a time-course assay to examine the correlation between uric acid accumulation and MICA/B expression. Both MICA/B expression and ROS levels was slightly increased at 4 and 8 hr but was boosted at 16 and 24 hr (Figure 9A and 9B). Uric acid started to accumulate at 8 hr but peaked at 16 hr in gemcitabine-treated cells and further increased in 5-FU-treated and irradiated cells at 24 and 32 hr (Figure 9C). 5-FU, gemcitabine, and irradiation induced uric acid accumulation in a dose-dependent manner (Figure 9C). Correspondingly, induction of ERK1/2 phosphorylation was detected at 16 hr in HT29 cells exposed to 5-FU, gemcitabine, and irradiation (Figure 9D). Induction of ERK1/2 phosphorylation in HT29 cells by 5-FU, gemcitabine, or radiation was also dose-dependent (Figure 9D).

### Uric acid accumulation is required for genotoxic stress-induced MAP kinase activation

**Figure 10:**
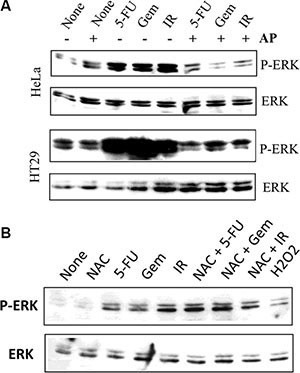
Allopurinol inhibits genotoxic stress-induced MAP kinase activation (**A**) Allopurinol inhibits MAP kinase activation. HeLa and HT29 cells were left untreated or pretreated with allopurinol (250 μg/ml) for 1 hr, followed by stimulation with gemcitabine (2 μM), 5-FU (10 μM), or radiation (40 Gy). After incubation for 24 hr, cells were harvested and analyzed for ERK1/2 phosphorylation and total ERK with an anti-ERK1/2 antibody. (**B**) The effect of NAC on genotoxic stress-induced ERK phosphorylation. HT29 cells were exposed to 5-FU (10 μM), gemcibatine (2 μM), irradiation (40 Gy) or H2O2 (100 μM) for 16 hr in the absence or presence of 10 mM NAC. ERK1/2 phosphorylation was analyzed by Western blot. Equal loading of the cell lysates was confirmed by probing with an anti-ERK1/2 antibody in a separate membrane.

We next tested whether inhibition of XOR activity by allopurinol led to the inhibition of ERK1/2 phosphorylation in tumor cells undergoing genotoxic stress. As shown in Figure 10A, ERK phosphorylation was induced in HeLa and HT29 cells when analyzed at 24 hr after exposure to 5-FU, gemcitabine, and radiation. Allopurinol completely blocked the induction of ERK1/2 phosphorylation in these cells (Figure 10A). We next tested if ROS had a role in genotoxic stress-induced MAPK activation. As shown in Figure 10B, NAC failed to suppress ERK phosphorylation in 5-FU- and gemcitabine-treated HT29 cells but partially inhibited the activation of EKR phosphorylation in HT29 cells exposed to radiation. Moreover, H_2_O_2_ (100 μM) weakly induced ERK phosphorylation in HT29 cells (Figure 10B).

### XOR plays an important role in genotoxic stress-mediated antitumor activity

**Figure 11:**
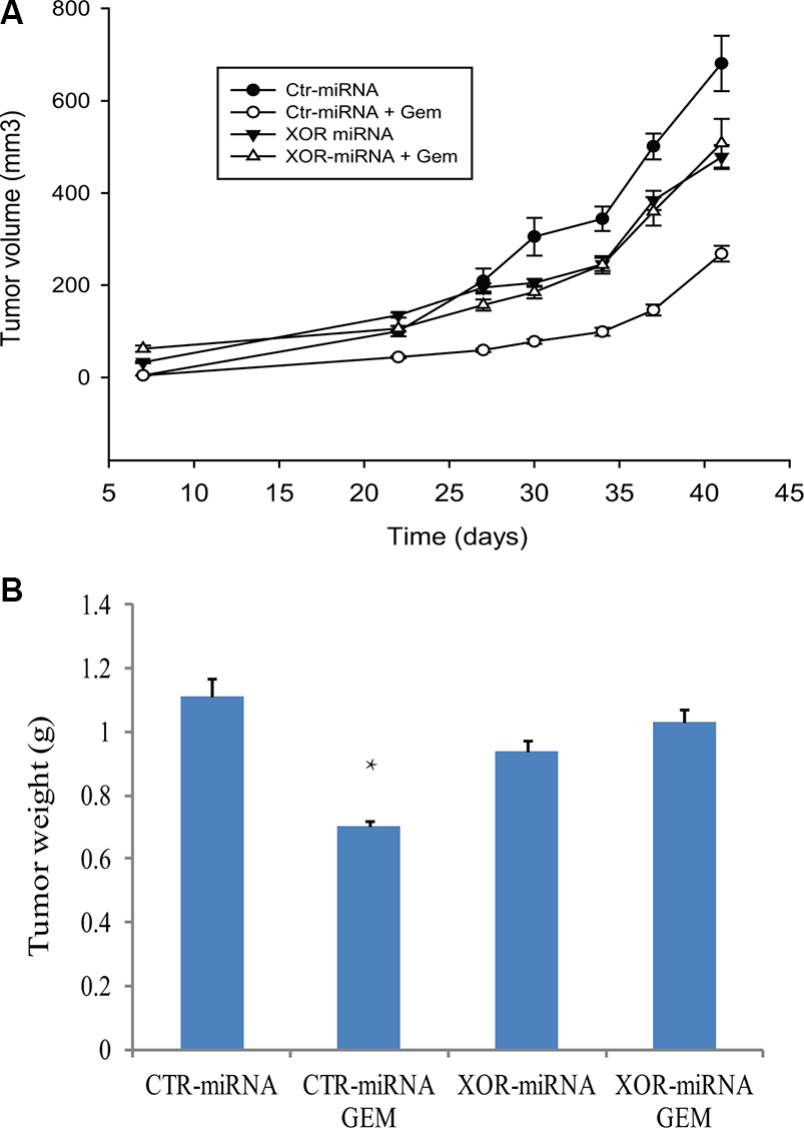
XOR knockdown ameliorates GEM-mediated antitumor activity (**A**) Differential effect of gemcitabine on the growth of Ctr-miRNA- and XOR-miRNA-transfected RCAS-Neu tumors. Female FVB mice (6-7-wks-old; 6 mice/group) were treated with saline or gemcitabine on day 1, 7, 14 days after intraductal injection of Ctr-miRNA- or XOR-miRNA-transfected cells (5 × 10^5^ cells/mouse). Tumor volumes were calculated and statistically analyzed. The growth of Ctr-miRNA-transfected tumors: saline vs. gemcitabine, *p* = 0.011; The growth of XOR-miRNA-transfected tumors: saline vs. gemcitabine, *p* = 0.501; The growth between Ctr-miRNA- and XOR-miRNA-transfected tumors, *p* = 0.056. (**B**) Differential effect of gemcitabine on tumor weight. Mice were sacrificed on day 42 after tumor cell injection. Tumors were collected and weighed. The differences in tumor weight between various groups were analyzed by using a Student *t* test. ** The *p value* compared to untreated control, < 0.05.

Finally we conducted an orthotopic mouse model of breast cancer by injection of RCAS-Neu cells into the fat pad of the mammary glands. In untreated mice, Ctr-miRNA-transfected RCAS-Neu tumors grew slightly faster than XOR-miRNA-transfected tumors. Statistical analysis revealed that there was no significant difference (*p* = 0.056) in tumor growth rate between Ctr-miRNA and XOR-miRNA-transfected tumors (Figure 11A). Gemcitabine treatment significantly inhibited the growth of Ctr-miRNA-transfected RCAS-Neu tumors, compared to saline treated group (*p* = 0.011). Surprisingly, gemcitabine treatment had no effect on the growth of XOR-miRNA-transfected RCAS-Neu tumors (Figure 11A). Gemcitabine treatment significantly decreased the weight of Ctr-miRNA-transfected RCAS-Neu tumors but had no effect on the weight of XOR-miRNA-transfected RCAS-Neu tumors (Figure 11B), compared to their untreated counterparts. This experiment was repeated twice with almost identical results.

## DISCUSSION

Our study provides several lines of evidence showing that uric acid production was responsible for the genotoxic stress-induced NKG2/D ligand expression: 1) Inhibition of XOR activity by allopurinol or XOR expression by XOR miRNA abrogated the genotoxic stress-induced NKG2D ligand expression, MAP kinase activation, and uric acid production; 2) Exogenous uric acid induced MICA/B expression; 3) Intracellular uric acid concentrations in MSU-treated cells were comparable to that in the cells exposed to genotoxic stress; 4) A375 cells that failed to uptake uric acid did not respond to MSU to induce MICA/B expression and to activate the MAP pathway. Of note, induction of MICA/B expression in HT29 cells undergoing genotoxic stress lagged behind uric acid accumulation. It makes sense since increased MICA/B expression was likely due to the transcriptional regulation mediated by AP-1 through the MAP kinase activation.

Mechanistic study revealed that genotoxic stress induced MICA/B expression by uric acid-mediated MAP kinase activation. Several lines of evidence support this supposition: 1) Exogenous MSU rapidly activated the MAP kinase pathway (Figure 7A); The inhibition of the MAP kinase pathway blocked MSU-induced MICA/B expression; 2) Inhibition of uric acid production by allopurinol in tumor cells undergoing genotoxic stress inhibited MAP kinase activation (Figure 10) and MICA/B expression (Figure 3); 3) We and others showed that RAS and BRAF oncogene mutation and activation leads to increased MICA/B expression [12, 14]; 4) The promoters of both the MICA and MICB genes contain a putative AP-1 site [18]. AP-1 is involved in regulating mouse NKG2D ligand gene expression [42]. It should be noted that MSU also activates other signaling molecules such as the proline-rich tyrosine kinase 2, p38 MAP kinase pathway, and NF-κB [43]. NF-κB induces MICA/B expression in activated T cells [44–46]. The signaling molecules and the transcription factors other than the MAP kinase pathway-activated AP-1, such as NF-κB, may also contribute to MSU-induced MICA/B gene expression.

While our data collectively suggest that uric acid produced by XOR plays a critical role in mediating genotoxic stress-induced NKG2D ligand expression, several questions remain to be answered: 1) it is not clear if MSU enters cells through endocytosis by binding the cell membrane lipids in a receptor-independent manner [47] or through uric acid transporter such as GLUT9 or URAT1 [48]; 2) The mechanisms by which increased concentrations of intracellular uric acid activate the MAP kinase pathway are not clear; 3) It is also not clear if uric acid produced in DNA-damaged cells can form precipitates to act like uric acid crystals.

Prior studies have shown that ROS induces MICA/B expression by activating the promoters of the MICA and MICB genes [17–20]. Another study showed that ROS induces MICB and ULBP1 expression in human airway epithelial cells, in part by increasing the transcripts of MICB and ULBP1 and by increasing the translocation of these NKG2D ligands to the cell surface [49]. ROS production is a well observed phenomenon in tumor cells exposed to genotoxic drugs and radiation. We found that ROS scavenging did not block the induction of MICA/B expression in cells exposed to 5-FU or gemcitabine but partially blocked the induction of MICA/B expression in these cells exposed to radiation at 16 hr (Figure 5). Exogenous hydrogen peroxide had only a minor effect on MICA/B expression (data not shown). These observations suggest that ROS production plays a minor or redundant role in inducing MICA/B expression in the tumor cell lines used in our study. This is particularly true that in the early phase of genotoxic stress, MICA/B expression was slightly increased in the absence of uric acid production whereas ROS levels were increased, which was independent of XOR (Figure 9). ROS may play a critical role in inducing NKG2D ligand expression in XOR-negative tumor cells undergone genotoxic stress.

Patients with XOR-negative breast and lung cancers are associated with a poor prognosis [28, 31]. Fini et al. [50] reported that two of three XOR inhibitors, oxypurinol and Y-700, but not allopurinol, modestly but significantly promote the growth of MDA-231 breast cancer in a xenograft mouse model. We found that XOR-miRNA-transfected RCAS-Neu tumors did not grow significantly slower than Ctr-miRNA-transfected RCAS-Neu tumors (Figure 11A). Interestingly, gemcitabine treatment inhibited the growth of Ctr-miRNA-transfected but not XOR-miRNA-transfected RCAS-Neu breast tumors (Figure 11B). We speculate that gemcitabine-mediated antitumor activity in this syngeneic breast cancer model is mediated by the induction of antitumor immunity through increased NKG2D ligand expression. In support of this notion, ectopic expression of the murine NKG2D ligands Rae1β or H60 in tumor cell lines results in potent rejection of the tumor cells [51]; Inhibition of XOR activity by allopurinol blocks tumor rejection in a syngeneic mouse model [35]. Moreover, a recent study showed that the shed form of MULT1, a high-affinity NKG2D ligand, activates NK cells and causes tumor rejection in part by competitively reversing a global desensitization of NK cells imposed by engagement of membrane NKG2D ligands on tumor-associated cells [52]. These observations collectively suggest that increased NKG2D ligand expression following genotoxic drugs or radiation and the subsequent antitumor immunity may contribute to their therapeutic effects.

In summary, our study showed that genotoxic stress-induced NKG2D ligand expression in tumor cell lines was largely mediated by increased intracellular uric acid concentrations through XOR. Uric acid induced NKG2D ligand expression by activating the MAP kinase pathway. XOR plays an important role in gemcitabine-mediated antitumor activity in a murine breast cancer model.

## MATERIALS AND METHODS

### Reagents and cell lines

Uric acid, allopurinol, 5- fluorouracil, and N-acetylcysteine (NAC) were purchased from Sigma (St. Louis, MO). MSU crystals were prepared as previously described [53]. Briefly, supersaturated uric acid solutions (4–5 mg/ml) in 0.1 M borate (pH 8.5) were left at room temperature for 72 h. The precipitate of MSU crystals were spin down and followed by washing with alcohol and acetone once. MSU crystals were air-dried and reconstituted in saline. SB202180 and PD98059 were purchased from Calbiochem (San Diego, CA). U026, anti-phospho ERK1/2, and anti-ERK1/2 antibodies were purchased from Cell Signaling Technology, Inc. (Beverly, MA). SP600125 was purchased from Creswood Technology Group (Yonker, NY). 2,7- dichlorodihydrofluorescin diacetate (DCF-DA) was purchased from Molecular Probes, Inc. (Eugene, OR). Gemcitabine (Gemzar, Eli Lilly, Indianapolis, IN) was purchased as a lyophilized powder and reconstituted in saline at the concentration of 130 μM. Radiation was conducted by exposing cells to a ^137^Cs resource in a CIS Biointernational generator at a rate of 1.45 Gy per minute. Anti-MICA/B mAb (Clones 6D4) was produced as previously described [3, 5, 6, 54]. FITC conjugated anti- HLA-ABC class I antibody (clone W6/32) were purchased from Serotec Ltd., (Oxford, UK). FITC conjugated anti-Pan-Rae1 antibody was purchased from R&D Systems. HT29, a human colon cancer cell line (originally MRO87 cells), was kindly provided by Dr. Kenneth B. Ain (University of Kentucky Medical Center, KY). HeLa (a human cervical cancer cell line) and A375 (a human melanoma cell line) cells were purchased from the American Tissue Culture Collection (Manassas, VA). RCAS-Neu cells are a murine breast cancer cell line that is derived from a breast cancer induced in TVA transgenic mice through intraductal injection of an avian retroviral vector encoding a mutant rat Neu oncogene [55]. RCAS-Neu and HT29 cells were grown in complete RPMI 1640 medium containing 10% fetal bovine serum. HeLa and A375 cells were grown in DMEM with 10% fetal bovine serum.

### RT-PCR

A forward primer 5- ATGCCCCAGTCC-TCCAGA GCTCAG-3 (start at nucleotide 453, A of the translation start codon ATG is designated as nucleotide 1) and a reverse primer 5′- GTGGCATCCCTGTGGTCACTCGTC- 3′ (end at nucleotide 1088) were used to amplify a 636- bp PCR fragment of the MICA gene. A forward primer 5′- GGCGTCAGGATGGGGTATCTTTGA- 3′ (start at nucleotide 716) and a reverse primer 5′- GGCAG GAGCAGTCGTGAGTTTGCC- 3′ (end at nucleotide 1429) were used to amplify a 714-bp PCR fragment of the MICB gene. Oligonucleotides 5′-TGAAGG- TCGGAGT CAACG-GATTTGGTC- 3′ and 5′- ATGGACTGTGGTC ATGAGTCC TTCCACG- 3′ were used to amplify a 527-bp GAPDH DNA fragment. The PCR reaction was set with an initial denaturation of 2 min at 94°C and subsequent denaturation for 45 s at 94°C, annealing for 45 s at 55°C, and extension for 1 min at 72°C. Thirty cycles were used to amplify the PCR product.

### XOR knockdown

The XOR hairpin DNA oliognucleotides were synthesized and cloned into a microRNA expression vector, pcDNA6.2/GW-miR according to manufacturer's instruction (Invitrogen, Carlsbad, CA). The sequences for oligonucleotides that target XOR mRNA were 5′- TGCTGTGATGATGTTCCCTCCAACGGGTTTT GGCCACTGACTGACCCGTTGGAGAACATCATCA- 3′ and 5′-CCTGTGATGATGTTCTCCAACGGGTCAGTCA GTGGCCAAAACCCGTTGGGGGAACATCATCAC- 3′. The sequences of the inserted oligonucleotides were verified by DNA sequencing. Two pairs of primers that target murine XOR RNA at nucleotide position 1261 and 1395 were cloned into the same vector. XOR miRNA targeting nucleotide position 1395 was used in whole study. The same expression vector encoding a miRNA sequence targeting β-galactosidase was included as a negative control. HeLa and HT29 cells were transfected with control or XOR miRNA and screened in the media containing blasticidin (10 μg/ml). Pools of stable colonies were analyzed for XOR expression by IF staining and Western blot.

### Immuofluorescence (IF) staining

XOR expression was detected by IF staining with a rabbit anti-XOR rabbit antibody (Rockland Immunologicals, Inc., Bertsville, PA) (1:100) and examined under a fluorescence microscope. The pictures were taken with a digital camera connected to Olympus BX41TF fluorescence microscope.

### Flow cytometric analysis

MICA/B expression was stained as previously described [12] and analyzed in a fluorescence-activated cell-sorter scanner (Becton Dickinson, Palo Alto, CA) and Cell Quest software.

### Quantification of uric acid

Cells were lysed in the buffer containing Tris-HCl, 50 mM, pH 8.0; 2 mM EDTA, 1% Triton X-100 and followed by a 10-second homogenization. The lysates were incubated on ice for 30 min and then spun down at 2000 g for 15 min. The supernatants were collected and analyzed for uric acid concentration by using a uric acid kit (BioAssay Systems, Hayward, CA). The difference in uric acid concentrations from the cells with different treatment was statistically analyzed by using an unpaired Student *t* test.

### ROS measurements

Cells were pretreated with 5-FU, gemcitabine, or radiation for the indicated lengths of time in the absence or presence of allopurinol or N-acytelcysteine (NAC) or gluthione which were added 1 hr prior to the exposure to MSU, 5-FU, gemcitabine, or radiation. Cells were harvested and then loaded with fluoroscein dye DCF-DA (20 nM) at 37°C for 15 min, followed by FACS analysis for intracellular ROS levels.

### Western blot

Cells were harvested and analyzed for MAP kinase activation by Western blot with an antibody against phosphor-ERK1/2. The membranes were stripped and reprobed with an anti-ERK1/2 antibody to ascertain equal loading of the cell lysates. XOR was detected with a rabbit antiserum (Lifespan BioScience, Inc., Seattle, WA). Actin was detected as a loading control with a monoclonal antibody (Santa Cruz Biotechnology, Inc., San Diego, CA).

### Orthotopic breast cancer model

The study was carried out in strict accordance with the recommendations in the Guide for the Care and Use of Laboratory Animals of the National Institutes of Health. The protocol was approved by the Institutional Animal Care and Use Committee of Rush University Medical Center. Female FVB mice (6–7-wks-old; 6 mice/group) were inoculated with Ctr-miRNA- or XOR-miRNA-transfected RCAS-Neu cells (5 × 10^5^ cells in 50 μl/mouse) by injection into the fat pad and treated with saline or gemcitabine (160 mg/kg body weight) by intraperitoneal injection on day 1, 7, 14 days. Tumor volumes were determined by caliper measurement, twice a week, and calculated based on the formula: length × width^2^ × π ÷ 6. Mice were sacrificed on day 42. Tumors were collected and weighed. The differences in tumor volumes were statistically analyzed by using the one-way repeated measure ANOVA. The differences in tumor weight were analyzed by using an unpaired Student *t* test. The *p value* of < 0.05 was considered statistically significant.

## Abbreviations

5-FU: 5-fluorouracil
AP: allopurinol
DCF-DA: 2,7-dichlorodihydrofluorescin diacetate
ERK: extracellular signal-regulated protein kinase
FACS: fluorescence-activated cell- sorter
Gem: gemcitabine
IR: ionizing radiation
MAP: mitogen-activated protein
MAPK: MAP kinase
MICA/B: major histocompatibility complex class I-related chain A and B
MHC: major histocompatibility complex
MSU: monosodium urate
NAC: N-acytelcysteine; NK natural killer
ROS: reactive oxygen species
XOR: xanthine oxidoreductase.
